# A Radiomics Approach to Assess High Risk Carotid Plaques: A Non-invasive Imaging Biomarker, Retrospective Study

**DOI:** 10.3389/fneur.2022.788652

**Published:** 2022-03-08

**Authors:** Sihan Chen, Changsheng Liu, Xixiang Chen, Weiyin Vivian Liu, Ling Ma, Yunfei Zha

**Affiliations:** ^1^Department of Radiology, Renmin Hospital of Wuhan University and Hubei General Hospital, Wuhan, China; ^2^Advanced Application Team, MR Research, GE Healthcare, Beijing, China; ^3^He Kang Corporate Management (SH) Co. Ltd, Shanghai, China

**Keywords:** carotid atherosclerosis, HRMRI, radiomics, symptomatic, asymptomatic

## Abstract

**Objective:**

This study aimed to construct a radiomics-based MRI sequence from high-resolution magnetic resonance imaging (HRMRI), combined with clinical high-risk factors for non-invasive differentiation of the plaque of symptomatic patients from asyptomatic patients.

**Methods:**

A total of 115 patients were retrospectively recruited. HRMRI was performed, and patients were diagnosed with symptomatic plaques (SPs) and asymptomatic plaques (ASPs). Patients were randomly divided into training and test groups in the ratio of 7:3. T2WI was used for segmentation and extraction of the texture features. Max-Relevance and Min-Redundancy (mRMR) and least absolute shrinkage and selection operator (LASSO) were employed for the optimized model. Radscore was applied to construct a diagnostic model considering the T2WI texture features and patient demography to assess the power in differentiating SPs and ASPs.

**Results:**

SPs and ASPs were seen in 75 and 40 patients, respectively. Thirty texture features were selected by mRMR, and LASSO identified a radscore of 16 radiomics features as being related to plaque vulnerability. The radscore, consisting of eight texture features, showed a better diagnostic performance than clinical information, both in the training (area under the curve [AUC], 0.923 vs. 0.713) and test groups (AUC, 0.989 vs. 0.735). The combination model of texture and clinical information had the best performance in assessing lesion vulnerability in both the training (AUC, 0.926) and test groups (AUC, 0.898).

**Conclusion:**

This study demonstrated that HRMRI texture features provide incremental value for carotid atherosclerotic risk assessment.

## Introduction

Carotid atherosclerotic plaques contribute to ~20% of the ischemic cerebrovascular events, including transient ischemic attack (TIA) (1). Clinical trials have demonstrated that ultrasonography-defined luminal stenosis of ≥70% was predictive of future ischemic events in both symptomatic (2, 3) and asymptomatic (4, 5) patients. However, numerous studies have demonstrated serious limitations of angiology-defined degree of luminal stenosis; therefore, carotid ultrasound screening in the general population is not recommended (6). An increasing number of studies have demonstrated the significance of different vulnerable plaques based on plaque compositions (7); for example, intraplaque hemorrhage (IPH) and a lipid-rich necrotic core (LRNC) have been recognized as high risk factors for stroke (8). There are many studies that focus on plaque as the major cause of disease, such as stroke and TIA, which are symptomatic. When finding risk factors, we need to differentiate the plaque of symptomatic patients from asymptomatic patients. Doppler ultrasonography (CDUS), computed tomography angiography (CTA), contrast-enhanced magnetic resonance angiography (MRA), and high-resolution magnetic resonance imaging (HRMRI) have been used for non-invasive prediction of carotid plaque recently. CDUS cannot proved three-dimensional (3D) information regarding the structures. CTA has the highest diagnostic performance, with the disadvantage of ionizing radiation (9). With the development of technology, the first-pass imaging of MRA could be used to analyze the vessel stenosis with good sensibility and specificity, especially for 70–90% stenosis (10, 11). Lower resolution and worse spatial resolution of the first-pass imaging of MRA are not suitable for predicting the structure of plaque. The acquisitions of non-isotropic voxels and lower matrix could result in partial volume effects that compromise carotid stenosis and plaque structure prediction (12). Meanwhile, steady-state imaging of MRA has greater accuracy and higher resolution in depicting the structure of the plaque (10). However, during the imaging of steady-state MRA, intravascular contrast agents could extend intravascular residence times with relatively increased cost. Gadobenate dimeglumine demonstrates feasibility in the diagnostic performance for carotid plaque (11). However, further research is needed to explore the feasibility of a conventional contrast agent of MRA.

In addition to the identification of lumen conditions, recent advanced HRMRI can characterize detailed features within the lesion structure, including the size of an LRNC, fibrous cap (FC) thickness, and the presence of IPH (13). These detailed morphological and compositional features have been demonstrated to be risk factors associated with patient clinical presentations (5, 14) and future ischemic events (15, 16). In most previous studies, plaque features were quantified in a straightforward way; for example, an LRCN was classified as small or large according to its size, and IPH was classified as being present or absent according to the signal intensity on T_1_-weighted images. However, atherosclerosis is a complex structure, with a mixture of fibrous tissue and lipids and different types of collagens (type I and type III) (16). The complexity of compositional features could be captured by *in vivo* imaging presenting a special image texture that has been studied the least. Pilot studies have demonstrated the clinical potential of image texture analysis. A coronary computed tomography angiography (CTA) radiomics-based machine learning model showed superior performance to that of expert visual assessment in the identification of advanced atherosclerotic lesions (17, 18). Histogram analysis with HRMRI provided significantly complementary values over luminal stenosis in defined lesion types for predicting intracranial atherosclerosis, and the dispersion of signal intensity was a particularly effective predictive parameter (19). Pilot studies have used high-dimensional texture features to aid clinical decision-making by comparing the difference between symptomatic and asymptomatic plaques without predicting the risk of plaques (20, 21).

This study aimed to build an effective model using a combination of HRMRI texture features and patient clinical risk factors to improve the accuracy in high-risk plaque identification, which would result in differentiation between symptomatic and asymptomatic plaques.

## Materials and Methods

### Patient Population

This study retrospectively recruited 323 patients with carotid plaques from July 2015 to May 2021 from the Renmin Hospital of Wuhan University. This study was approved by the local ethics committee, and the patients provided signed informed consent. All the patients had at least 30% carotid luminal stenosis, as defined by ultrasound angiography and Doppler ultrasonography (22). All the patients were divided into symptomatic plaque (SP) and asymptomatic plaque (ASP) groups. The diagnostic principle of an SP is as follows: (a) an acute ischemic stroke within the last 7 days in patients who had a corresponding unilateral infarct restricted to the territory of a single carotid artery defined by diffusion-weighted imaging (23); (b) patients with a symptom duration of ≤ 24 h who had met the World Health Organization definition of transient ischemic attack but had a documented acute ischemic infarct; (c) carotid luminal stenosis >30% (24), and (d) thickness of plaques confirmed to be larger than 2 mm (25).

The exclusion criteria were as follows: (a) patients with carotid artery stenosis ≥70%, (b) cardiogenic stroke, (c) patients with bilateral infarct or clinical symptoms caused by bilateral carotid plaque, and (d) other reasons, such as poor image quality of HRMRI. Finally, 208 patients were excluded from the study.

### HRMRI and Analysis

#### HRMRI Scanning Protocol

HRMRI was performed using a 3.0T MR750 system (GE Healthcare, United States) with an 8-channel carotid coil (GE Healthcare, United States). The protocol of HRMRI included (1) T1 weighted MRI, (2) T2 weighted MRI, (3) proton density (PD) weighted MRI, and (4) 3D time of flight MRI (3D TOF), matrix = 512^*^512, slice thickness = 2 mm.

Two-dimensional (2D) T1-weighted double inversion recovery fast spin echo (FSE), repetition time/echo time (TR/TE) = 800/7.5 ms, flip angle = 107°, acquisition = 2, bandwidth (BW) = 122.07 Hz, and echo train length = 12, matrix = 512^*^512, slice thickness = 2 mm.

PD-weighted FSE, TR/TE = 1,558/29.5 ms, flip angle = 107°, acquisition = 1, and BW = 81 Hz; 2D T2-weighted double inversion recovery FSE, TR/TE = 1,578/69 ms, flip angle = 107, acquisition = 1, and BW = 81 Hz, matrix = 512^*^512, slice thickness = 2 mm.

3D-TOF, TR/TE = 23/5.7 ms, flip angle = 22°, acquisition = 2, and BW = 280 Hz, matrix = matrix = 512^*^512, slice thickness = 2.6 mm.

#### Robustness of Feature

**1) Imaging Preprocessing**

Before radiomics extraction, all the original images were pre-processed using normalization schemes through MATLAB (version 2016a). Since the intensity of the signal in MRI is not considered an absolute value but is relative to the technical parameters, the images underwent standardization and were normalized. The standardization equation is as follows:


$$\begin{array}{l} image\;standardization=\frac{x\;-\;\mu }{adjusted\;<uscore>\;stddv}\\ \;adjusted_{stddev}\;=\;max\;(\sigma ,\frac{1.0}{\sqrt{}N}) \end{array}$$

where μ indicates the mean value of the signal in images, x indicates the matrix, σ indicates the standard deviation, and N indicates the number of voxels.

The normalized equation is as follows:


$$\begin{array}{l} normalize=\frac{x_{i}-\;min\;(x)}{\max\;(x)-\;min\;(x)} \end{array}$$

X_i_ indicates the signal of the voxel. According to the standardized workflow of radiomics, images should be preprocessed before radiomics features extraction (26).

**2) Image Quantization**

Image quantization means the conversion of the gray values of the images to a discrete group of gray values (27). Before radiomic feature extraction, we quantized the image with a fixed bin width of 5. This value was chosen based on the example setting of Pyradiomics (https://github.com/AIM-Harvard/pyradiomics/tree/master/examples/exampleSettings). According to the protocol, the ideal number of bins is between 16 and 128 bins (28). A method to set a suitable bin width is to extract the feature named first order range such that it remains approximately in this bin range. The results of the range are presented in Supplementary Material 1. An absolute discretization was performed with fixed bin size (binsize = 5) such that the new bin had been assigned to pixel intensities to each BS gray level starting from 0. The equation is as follows:


$$\begin{array}{l} I_{BS}\left(x\right)=\left[\frac{I\left(x\right)}{BS}\right]-min\left(\left[\frac{I\left(x\right)}{BS}\right]\right)+1 \end{array}$$

I(x) indicates the intensity of the voxel; BS (binsize) indicates the bin size and I_BS_(x) indicates the gray level of voxel x that had been discretized (29).

All the images were transformed into Gaussian and wavelets. Wavelet filtering yields eight decompositions per level. The settings of the wavelet filter were as follows:

1) start_level [0]: integer, 0 based level of wavelet, which should be used as the first set of the decompositions from which a signature is calculated. 2) level [1]: integer, number of levels of wavelet decompositions from which a signature is calculated. 3) wavelet [“coif1”]: string, type of wavelet decomposition. Enumerated value, validated against possible values present in the pyWavelet.wavelist(). Current possible values (pywavelet version 0.4.0). Gaussian image is obtained by convolving the image with the second derivative (Laplacian) of a Gaussian kernel. The Gaussian kernel is calculated as follows:


$$\begin{array}{l} G\left(x,y,z,\sigma \right)=\frac{1}{{\left(\sigma \sqrt{}2\pi \right)}^{3}}e^{-\frac{x^{2}+y^{2}+z^{2}}{2\sigma^{2}}} \end{array}$$

The Gaussian kernel was convolved by the ∇^2^*G*(*x, y, z*) (30).

**3) ROI Segmentation**

T2WI was segmented manually using ITK-SNAP (version 3.6.0 www.itksnap.org). The regions of interest (ROI) were manually segmented by the max plaque. The ROIs of the plaques were delineated manually by two radiologists with 5 and 10 years of experience who were blinded to the clinical information. Because of the same matrix, the ROI would be used to extract the radiomics features for T1WI and PDWI (proton density weighted image). Then, the radiomics features were extracted from the ROI segmented by radiologists with 5 years' (Sihan Chen) and 10 years' (Yunfei Zha) experience to calculate the inter-observer correlation coefficients. Then, the radiologist with 10 years' experience (Yunfei Zha) would segment the ROIs twice after 4 weeks.

**4) Radiomics Feature Extraction**

**Figure 1:**
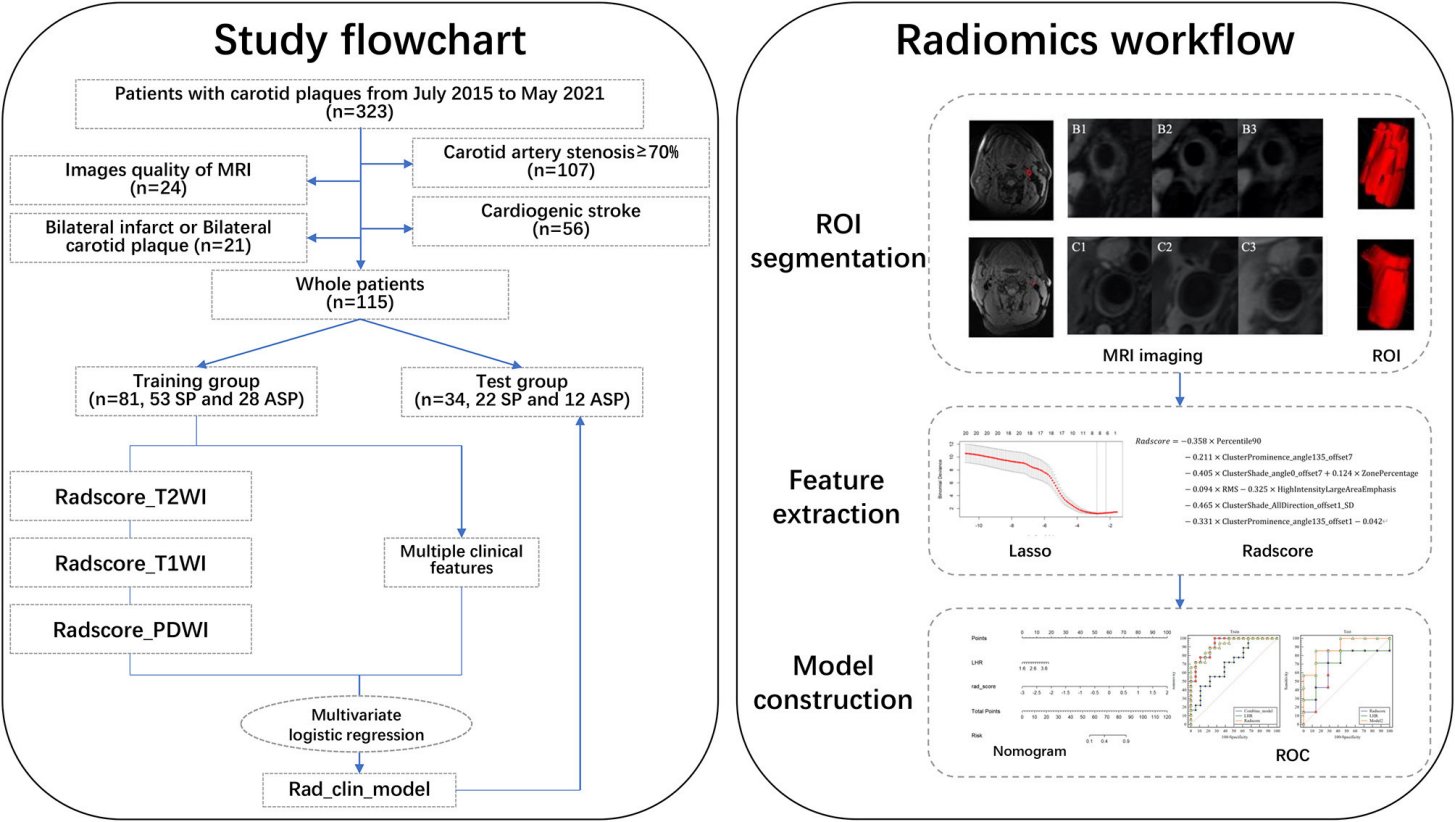
Study flowchart and Raidomics workflow.

The radiomics features were extracted using Anaconda Prompt (version 4.2.0) importing the feature package of pyradiomics (github.com/Radiomics/pyradiomics), according to the feature guidelines of the Image Biomarker Standardization Initiative (IBSI) (Figure 1A). Before radiomics extraction, all the images were co-registered, normalized, and resampled to 1 ×1 ×1 mm^3^ for an interpolation step. The radiomics features were extracted using Python (version 3.9.6) by importing the feature package of pyradiomics (github.com/Radiomics/pyradiomics) according to the feature guidelines of the IBSI (20). First-order features, shape features, gray-level co-occurrence matrix (GLCM), gray-level run-length matrix (GLRLM), gray-level size zone matrix (GLSZM), and gray-level size zone matrix (GLDM)-based original images and Gaussian and wavelet images were extracted.

**5) Inter-Observer Correlation Coefficient and Intraclass Correlation Coefficient**

Subsequently, the intraclass correlation coefficient was calculated by the radiologist with 10 years' experience using the two segmented ROIs. The features maintained an inter-observer correlation coefficient ≥ 0.7 and intraclass correlation coefficient ≥ 0.7 (31, 32). The values of the ICC ranged from 0 to 1. After calculation, the radiomics features were differentiated into three levels: poor robustness, with ICC values <0.5; moderate robustness, with 0.5 < ICC values <0.9, and excellent robustness, with ICC values ≥ 0.9. (Supplementary Materials 2).

### Radiomics Signature Construction

The initial feature selection maintained an inter-observer correlation coefficient and intraclass correlation of ≥0.7. Feature reduction was then performed using training data with the minimum redundancy maximum relevance (mRMR) algorithm to find a set of features S with n features {*X*_i_} that maximally depend on the target label (17). The max-dependency is computed as


$$\begin{array}{l} \text{maxD}\left(\text{S},\text{c}\right),\text{D}=\text{I}\left(\left\{{\text{x}}_{\text{i}},\text{i}=1,...,\text{n}\right\};\text{c}\right) \end{array}$$

At the same time, when n>1, we should provide a set of n-1 features, Sn-1; the set can be calculated as the leading contribution to the largest increase in I(S; c),


$$\begin{array}{l} \text{I}(\text{S};\text{c})\text{ = }\int \cdot \cdot \cdot \int \text{p}({\text{x}}_{1},\;...,{\text{ x}}_{\text{n}},\text{ c})\text{log}[\text{p}({\text{x}}_{1},\;...,{\text{ x}}_{\text{m}},\text{ c})\\ \;/p(x_{1},\;...,\;\;x_{\text{m}})p(c)]dx_{1}\cdot \cdot \cdot dx_{\text{m}}dc \end{array}$$

The mRMR calculates a set of features by maximizing the difference and minimizing redundancy. After feature selection, the LASSO algorithm was selected to build the radiomics signature by optimizing the subset of features to construct the final model. Radiomics score (Radscore) was applied to build a diagnostic model to discriminate between the stable and unstable plaques based on the training set. After feature elimination, the remaining final features were used to build the radiomics signature via a linear combination of selected features that were calculated by the respective coefficients of each feature (17).

### Development of the Nomogram

Clinical variables in the training group were tested in a multivariate logistic regression model to differentiate the ASPs and SPs. The variance inflation factor (VIF) was calculated to predict the collinearity of the clinical variable. The clinical variable was maintained with VIF ≤ 5 and used to build the clinical model using the multivariate logistic regression model-based minimum AIC. The Rad_clin model combined Radscore and clinical variable were subsequently tested in a multivariate logistic regression model to differentiate the SPs and ASPs. A nomogram would be constructed based on the Rad_clin model.

### Model Effectiveness Evaluation

The diagnostic performance of the radiomics signature, clinical model, and combined model have been compared by the area under the receiver operating characteristic (ROC) curve (AUC) both in the training group and validated by the test group, each person of Radscore had been calculated using the formula built in the training group. The accuracy of the radiomics signature was evaluated for both the training and test groups. The calibration of the models was assessed using calibration curves and the Hosmer–Lemeshow test; decision curve analysis (DCA) was performed to estimate the clinical utility of the models.

### Statistical Analysis

Statistical analysis was performed using R 3.6.1 (www. Rproject.org). The packages in R used in this study were tidyverse, caret, pROC, glmnet, DMWR, rmda, ggpubr, ModelGood, rms, mRMRe, DescTOOLs, and irr. mRMR were used for feature reduction to avoid overfitting. After mRMR, LASSO with 10-fold cross-validation was used to construct a radiomics signature (Radscore). During the clinical model construction, the clinical variable had been excluded if the variance inflation factor (VIF) ≥5, and the clinical variable could be subsequently maintained using a *p* <0.05 during the single factor logistic regression. After that, the clinical variable in the clinical model would been kept based on minimum AIC (Akaike information criterion) principle. The combined model, based on the clinical variable and Radscore, and also the clinical model would be constructed by multiple logistic regression. The Delong test was applied to compare the differences in the ROC curves between the two arbitrary models using Medcalc (www. medcalc.org). The multiple comparisons would be corrected by Bonferroni's method. The differences in demographic and clinical variables were compared between patients with SPs and ASPs and in both the training and test group using GraphPad Prism 8 (www.graphpad-prism.cn). The Mann-Whitney *U*-test was used for non-normally distributed quantitative data, while the independent sample *t*-test was used for normally distributed data. Chi-squared tests were used to analyze the categorical data.

## Results

### Patient Clinical Characteristics

The study flowchart is presented in Figure 1. HRMRI from 115 patients, who had 75 SPs and 40 ASPs, was used for the final analyses. All the patients were divided into training and test groups in a ratio of 7:3. The patient demographics of the groups are listed in Table 1. These clinical variables showed no significant differences between the training and test groups.

**Table 1:** Clinicopathological characteristics of patients in the training group and test group.

**Characteristics**	**Training set (*n =* 81)**	**Test set (*n =* 34)**	***P-*values**
	**No. (70%)**	**No. (30%)**	
Gender			1.000
Male	64 (79.0)	27 (79.4)	
Female	17 (21.0)	7 (20.6)	
Age, year	51.2 ± 13.8	51.8 ± 12.2	0.815
**Laboratory examination**
LDL (mmol/L)	3.4 ± 0.3	3.4 ± 0.3	0.952
HDL (mmol/L)	1.2 ± 0.2	1.2 ± 0.2	0.487
LHR	3.0 ± 0.6	2.9 ± 0.5	0.452
**Medical history**
Hypertension	66 (81.5%)	23 (67.6%)	0.169
Diabetes	33 (40.7%)	8 (23.8%)	0.1223
**Plaque composition**
IPH	31	12	0.928
LRNC	40	18	0.887

*Age, LDL, HDL, and LHR are presented as mean ± standard deviation*.

*LDL, low-density lipoprotein; HDL, high-density lipoprotein; LHR, LDL/HDL ratio; IPH, intraplaque hemorrhage; LRNC, lipid-rich necrotic core*.

### Radiomics Signature Constructed for the Carotid Plaque

**Figure 2:**
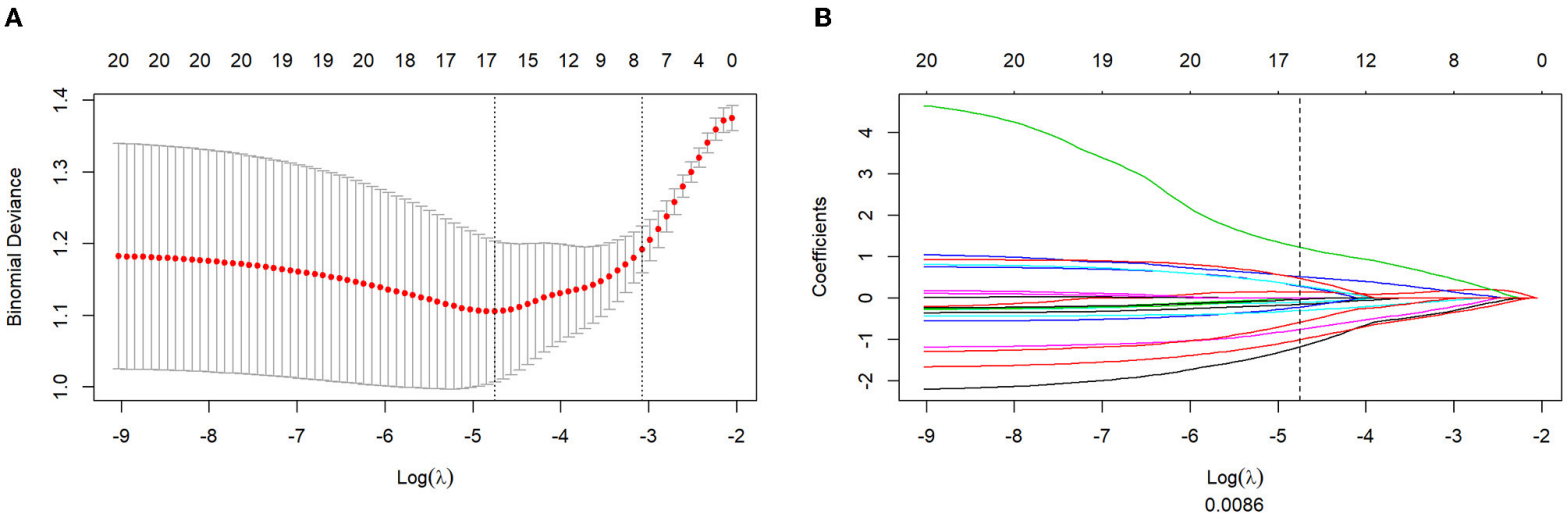
Texture feature selection using the absolute shrinkage and selection operator (LASSO) binary logistic regression model. **(A)** The penalty term (λ) in the LASSO model was selected through 10-fold cross-validation which is based on minimum criteria. Y-axis means binomial deviances. Down-X-axis means the log (λ), Up-X-axis means the average number of predictors. The red dots indicate average deviance values for each λ that different models have different deviance. Left-vertical-line shows the lowest deviance that the model showed the best fit to our training group. **(B)** LASSO coefficient profiles of the 16 features. The dotted vertical line was drawn at the value where log λ (0.008) resulted in eight non-zero coefficients.

A total of 1,121 features were initially extracted from T2WI. For the intraclass correlation coefficient, 309 features (27.5%) showed excellent robustness, 150 (13.3%) features showed poor robustness, and 662 (59.2%) features showed moderate robustness. After the mRMR operation, 30 features were retained by LASSO for both weightings. The log λ (0.0086) (Figure 2A) identified 16 (Figure 2B) for the identified eight features of the T2WI. The formulation of radiomics signature (Figures 2A,B) for T_2_-weighting is as follows:


$$\begin{array}{l} Radscore\text{ = −1.182 × }wavelet\_LL\_glszm\_ZoneEntropy\\ \text{ − 1.008 × }wavelet\_LH\_firstorder\_RootMeanSquared\\ \text{ + 0.284 × }wavelet\_HH\_glcm\_ClusterShade\\ \text{ + −0.108 × }wavelet\_HL\_firstorder\_RootMeanSquared\\ \text{ + −0.756 × }wavelet\_HL\_firstorder\_Skewness\\ \text{ + −0.153 × }wavelet\_HH\_firstorder\_Median\\ \text{ + 0.141 × }original\_ngtdm\_Busyness\\ \text{ + 1.224 × }wavelet\_HL\_firstorder\_TotalEnergy\\ \text{ + −0.218 × }original\_shape2D\_Elongation\\ \text{ + −0.304 × }wavelet\_LH\_firstorder\_Skewness\\ \text{ + −0.578 × }wavelet\_LL\_glcm\_ClusterShade\\ \text{ + −0.028 × }wavelet\_LH\_glcm\_Correlation\\ \text{ + 0.519 × }original\_glrlm\_ShortRunLowGrayLevelEmphasis\\ \text{ + 0.301 × }wavelet\_HH\_glcm\_Correlatio\text{n}\\ \text{ + −0.039 × }original\_shape2D\_Sphericity\\ \text{ + 0.472 × }wavelet\_HH\_glszm\_GrayLevelNonUniformity\\ \text{ + 0.569} \end{array}$$

**Figure 3:**
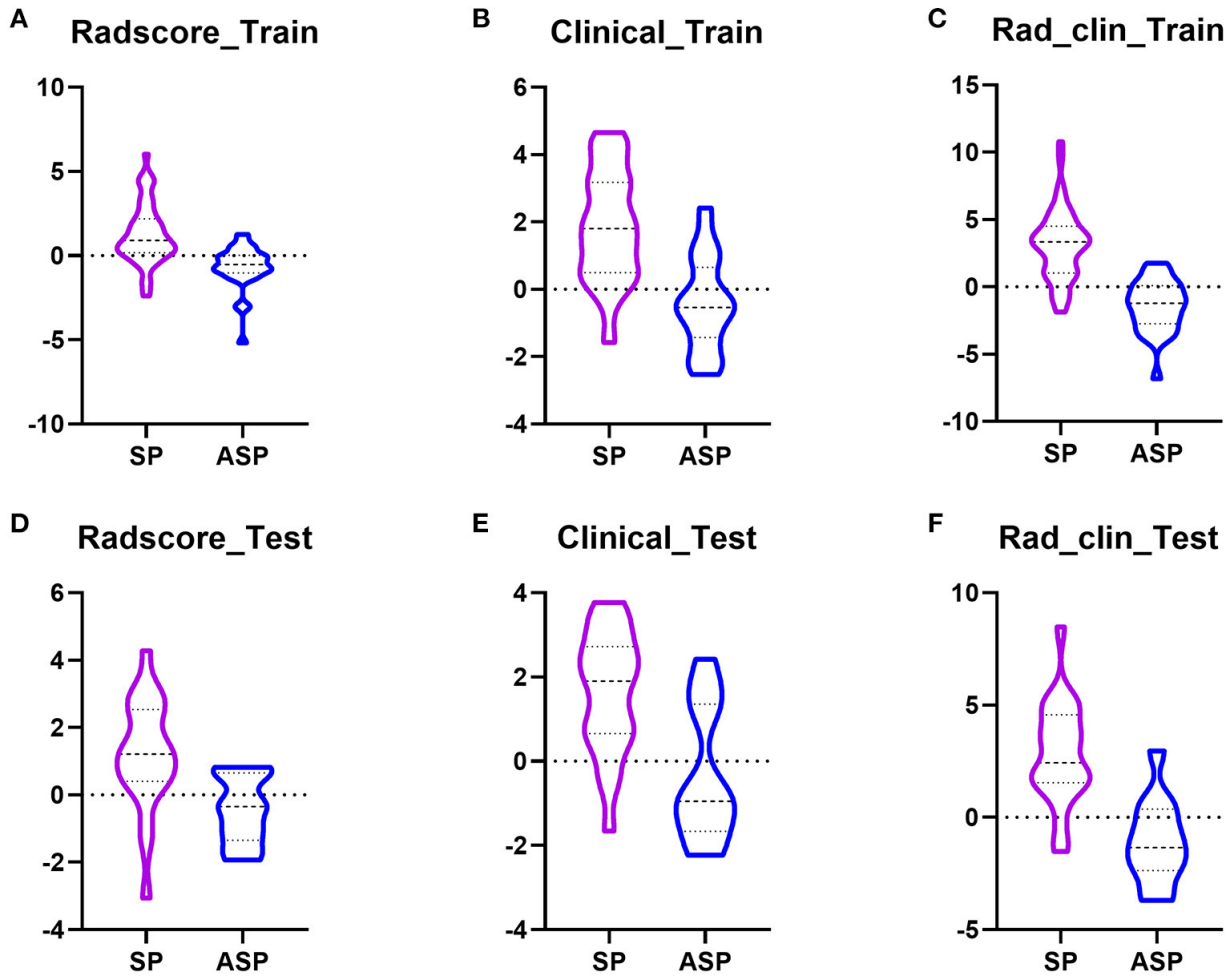
Boxplots showed the Radscore, clinical model, and Rad_clin model value of SPs and ASPs in the training and test groups. **(A,D)** Radscore in the training group and the test group; **(B,E)** Radscore in the training group and the test group; **(C,F)** Radscore in the training group and the test group.

The Radscore based on the T2WI of SPs was significantly higher than that of ASPs, in both the training (*p* = 0.0001, Figure 3A) and test groups (*p*=0.0018) (Figure 3D). At the same time, there were no significant differences in the Radscore between the training group and test group (*p* = 0.6458) which means there was no overfitting in the Radscore. The AUC of the Radscore in the training group and test group were 0.834 and 0.818.

Radscore_T1WI has been built based on T1WI (logλ = 0.070) and Radscore_PDWI based on PDWI (logλ = 0.043) (Supplementary Figure S1). The Delong test was conducted to analyze the difference in the diagnostic performance for Radscore in the training and test groups. There were no significant differences between Radscore_T2WI and Radscore_T1WI in the training group (*P* = 0.349) and test group (*P* = 0.502). At the same time, there were no significant differences between Radscore_T2WI and Radscore_PDWI in the training group (*P* = 0.492) and test group (*P* = 0.731). Radscore_T2WI was used to build the combined model for the highest AUC.

### Clinical Model Construction to Predict Carotid Plaques

Patients were randomly divided into training and test groups, with 53 patients with SP and 28 with ASP in the training group and 22 patients with SP and 12 with ASP in the test group. In the training group, the laboratory examinations of low-density lipoprotein (LDL) (*p* = 0.013), high-density lipoprotein (HDL) (*p* = 0.000), and LDL/HDL ratio (LHR) (*p* = 0.000) were significantly different between the patients with SP and ASP (Table 2). The plaque composition, IPH (*p* = 0.0001), and LRNC (*p* = 0.001) were significantly different between patients with SP and ASP. Age (*p* = 0.001) was significantly different between the SPs and ASPsn the test group. LDL (odds ratio [OR] = 9.10), HDL (OR = 0.019), LHR (OR = 5.31), IPH (OR = 9.33), and LRNC (OR = 6.56) were maintained owing to a variance inflation factor (VIF) ≤ 5. LHR, IPH, and LRNC were used to build a clinical model and a combined model based on the minimal AIC principle. LHR, IPH, and LRNC were used to construct a clinical model and the Rad_clin_model was constructed combined with the Radscore. The clinical model (*p* = 0.003 vs. *p* = 0.007, Figures 3B,E) and Rad_clin_model (*p* = 0.004 vs. *p* = 0.0001, Figures 3C,F) showed significant differences between SPs and SAP ASPs in the training and test groups. After correction by Bonferroni's analysis, the clinical model (*p* = 0.009 vs. *p* = 0.021) and Rad_clin_model (*p* = 0.012 vs. *p* = 0.0003) also showed significant differences between the SPs and ASPs in the training and test groups.

**Table 2:** Clinicopathological characteristics of APs and ASPs in a training group and a test group.

**Characteristics**	**Training set (*****n** **=*** **81)**		***P-*values**	**Test set (*****n** **=*** **34)**		***P-*values**
	**SP**	**ASP**		**SP**	**ASP**	
	***n =* 53**	***n =* 28**		***n =* 22**	***n =* 12**	
Gender			1.000			1.000
Male	42	22		17	10	
Female	11	6				
Age, year	52.9 ± 12.4	48.0 ± 15.9	0.123	47.5 ± 9.6	59.8 ± 12.9	0.001
**Laboratory examination**
LDL (mmol/L)	3.5 ± 0.2	3.3 ± 0.3	0.013*	3.5 ± 0.2	3.4 ± 0.3	0.529
HDL (mmol/L)	1.1 ± 0.2	1.3 ± 0.2	0.000*	1.2 ± 0.2	1.3 ± 0.2	0.222
LHR	3.2 ± 0.5	2.7 ± 0.6	0.000*	3.0 ± 0.4	2.8 ± 0.6	0.209
**Medical history**
Hypertension	43	23	1.000	16	7	0.635
Diabetes	20	13	0.603	6	2	0.784
**Plaque composition**
IPH	28	3	0.000*	12	0	0.005
LRNC	34	6	0.001*	14	4	0.183

*SP, symptomatic plaque; ASP, asymptomatic plaque; LDL, low-density lipoprotein; HDL, high-density lipoprotein; LHR, low-density lipoprotein; IPH, intraplaque hemorrhage; LRNC, lipid-rich necrotic core*.

### Diagnostic Performance of the Radscore, Clinical Model, and Rad_Clin Model

**Figure 4:**
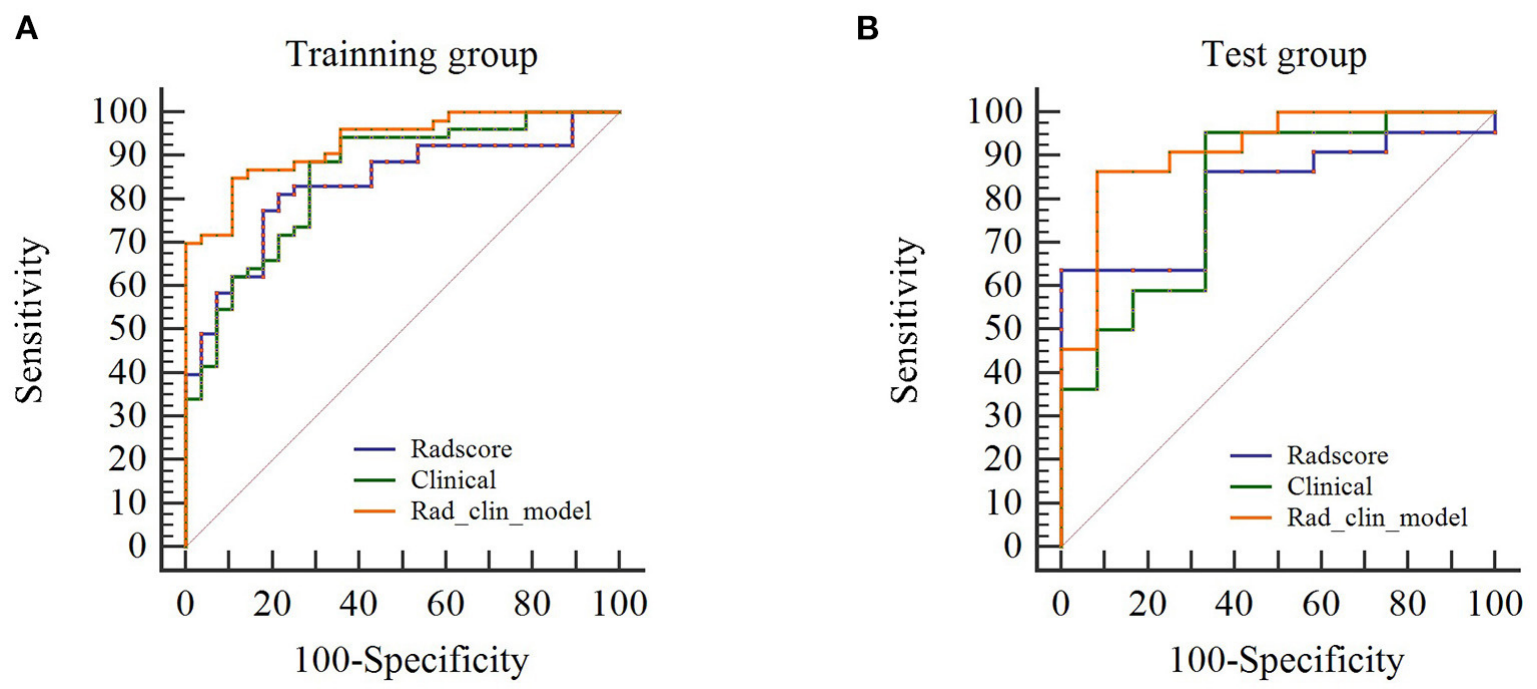
Diagnostic performance of univariate logistic regression model. **(A,B)** show the receiver operating characteristic curves of the Rad_clin model that show that the area under the curve is the highest among the Radscore and clinical model in the training and testing groups.

We also evaluated the discriminatory efficiency of the clinical model and the Rad_clin model using ROC analyses (Table 3, Figures 4A,B). The Rad_clin model yielded the largest AUC of 0.929 (95% confidence intervals [CI], 0.881–0.982) in the training group and 0.912 (95% CI, 0.810–1.000) in the test group, which showed significant differences between the clinical model (*p* = 0.023) and Radscore (*p* = 0.013) in the training group, but not in the test group (*p* = 0.090 vs. *p* = 0.155). The Radscore was not significantly different from the clinical model in both the training group (*p* = 0.782) and the test group (*p* = 0.852). The Hosmer-Lemeshow test in the Rad_clin model showed no significant differences in the goodness-of-fit for the training group (*p* = 0.454) and test group (*p* = 0.7442).

**Table 3:** Diagnostic performance of the Radscore, clinical model, and Rad_clin_model.

	**Radscore**		**Clinical**		**Rad_clin_model**	
	**Training group**	**Test group**	**Training group**	**Test group**	**Training group**	**Test group**
AUC	0.834	0.818	0.852	0.818	0.929	0.912
Accuracy	0.802	0.794	0.827	0.794	0.864	0.882
**95% CI**
Lower	0.753	0.674	0.771	0.662	0.881	0.810
Upper	0.942	0.962	0.943	0.971	0.982	1.000
Sensitivity	0.811	0.863	0.887	0.864	0.849	0.950
Specificity	0.786	0.667	0.714	0.667	0.893	0.786
PPV	0.877	0.826	0.854	0.826	0.937	0.864
NPV	0.687	0.727	0.769	0.727	0.757	0.917

*AUC, area under the curve; 95%CI, 95% confidence interval; PPV, positive predictive value; NPV, negative predictive value*.

### The Evaluation of the Rad_Clin Model

**Figure 5:**
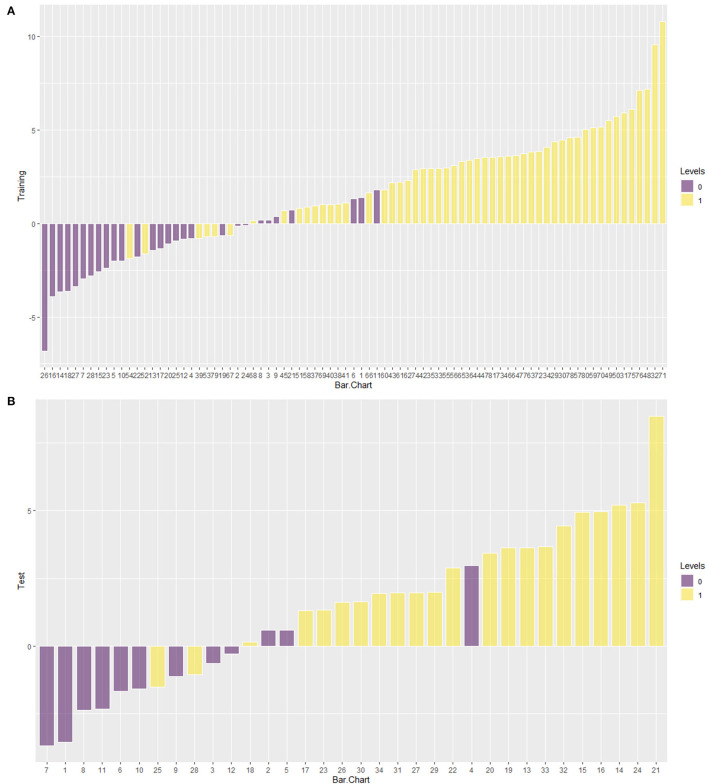
Barchart of the Rad_clin model in the training group and the test group. Yellow means symptomatic plaque (SP) and purple means asymptomatic plaque (ASP). When purple appears in the yellow area, this means the SP patients had been incorrectly predicted as ASP. When yellow appears in the purple area, this means the SP patients had been incorrectly predicted as ASP. **(A)** was the training group, **(B)** was the test group.

**Figure 6:**
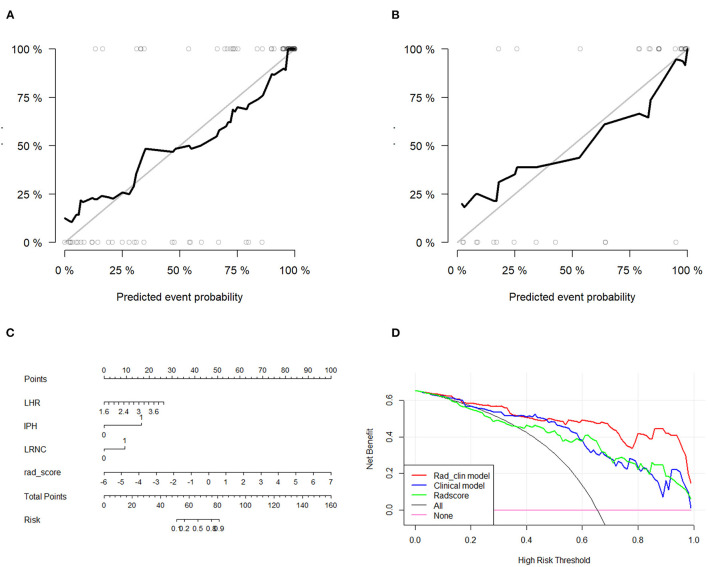
Diagnostic performance evaluation in the training group and test group. Calibration curves of the nomogram in the training **(A)** and the test groups **(B)**. Calibration curves showed the calibration of the nomogram in terms of agreement between the predicted risk of vulnerable plaque and high-resolution magnetic resonance imaging (HRMRI)-observed vulnerable plaque. **(C)** Nomogram based on Rad_clin_model. **(D)** Decision curves for Radscore, clinical model, and Rad_clin_model; the Y-axis shows the model benefit. The red line represents the Rad_clin_model. The blue line represents the clinical model, and the green line represents the Radscore. The X-axis means the threshold probability. ROC, receiver operating characteristic; AUC, area under the curve.

After the Rad_clin model was constructed, each patient in the training group (Figure 5A) and the test group (Figure 5B) was analyzed based on whether they had SP, and the patients who were incorrectly assessed are shown in Figure 5. Calibration curves of the nomogram in the training (Figure 6A) and the test groups (Figure 6B) showed good calibration of the nomogram in terms of agreement between the predicted risk of vulnerable plaque and high-resolution magnetic resonance imaging (HRMRI)-observed vulnerable plaque. The nomogram based on the Rad_clin model is shown in Figure 6C. The equation of the nomogram is as follows:


$$\begin{array}{l} Risk=-5.489+LHR\times 1.602+IPH\times 2.423\\ \;+LRNC\times 1.353+Radscore\times 1.122 \end{array}$$

The value of IPH defined 1 as patients with IPH. Meanwhile, the value of LRNC defined 1 as patients with LRNC. The DCAs for the Rad_clin model are shown in Figure 6D. The DCA indicated the threshold probability, in the range of 0 to 1, indicating that patients with carotid plaques could benefit from the Rad_clin model.

## Discussion

In this study, we extracted high-throughput imaging texture features from T2WI of carotid atherosclerotic plaques to build a multivariable logistic regression model with the combination of patient clinical risk factors to differentiate SPs and ASPs, which had the highest diagnostic performance to assess SPs. On the other hand, joint analysis of radiomics and clinical features could be of great significance in the differential diagnosis of other indistinguishable diseases.

This study demonstrated that the SP of symptomatic carotid plaques could be assessed using a T2WI-based radiomics model, constructed using a radiomics signature and clinical data. Traditionally, lesion vulnerability was assessed according to geometric parameters, such as stenosis (33), the size of the LRNC, the thickness of the fibrous cap (34, 35), and the presence of inflammation and IPH. In addition to HRMRI, we selected age, sex, LDL, HDL, hypertension, diabetes, and plaque composition as clinical factors to evaluate SPs, of which LHR, IPH, and LRNC had been integrated into the combined model to demonstrate the high-risk factors for cardiovascular diseases (22, 36–39). Clinical model-based LHR, IPH, and LRNC did not have a significantly better performance than the radiomics model in both the training and testing groups. However, the combination of LHR, IPH, LRNC, and radiomics could better predict the SPs than any of them alone.

There are16 radiomics features, namely, wavelet_LL_glszm_ZoneEntropy, wavelet_LH_firstorder_RootMeanSquared, wavelet_HH_glcm_ClusterShade, wavelet_HL_firstorder_RootMeanSquared, wavelet_HL_firstorder_Skewness, wavelet_HH_firstorder_Median, original_ngtdm_Busyness,wavelet_HL_firstorder_TotalEnergy, original_shape2D_Elongation, wavelet_LH_firstorder_Skewness, wavelet_LL_glcm_ClusterShade, wavelet_LH_glcm_Correlation, original_glrlm_ShortRunLowGrayLevelEmphasis,wavelet_HH_glcm_Correlation,original_shape2D_Sphericity, wavelet_HH_glszm_GrayLevelNonUniformity, extracted from T2WI, showed maximum significance. ZoneEntropy measures the randomness in the distribution of the zone sizes and gray levels. Root mean squared is the square-root of the mean of all the squared intensity values. Skewness measures the asymmetry of the distribution of the values of the mean value of the gray level. Median indicates the median value of the gray level intensity in the ROI. Busyness measures the change from a pixel to its neighbor. Total energy measures the value of energy feature scaled by the volume of the voxel in cubic mm. Elongation measures the relationship between the two largest components in the ROI shape. Cluster shade measures the skewness and uniformity. Correlation measures the value between 1 and 1, which demonstrates linear dependency of the gray level to the respective voxel. Sphericity measures the roundness of the shape of the ROI relative to a sphere. Gray level non-uniformity measures the variability of the gray level values in the ROI. When constructing the Radscore to diagnose SPs based on T2WI, its diagnostic performance reached 0.834 in the training group and 0.818 in the test group. The diagnostic performance was similar to the study by Zhang et al. (40). The importance of using standardized imaging protocols to eliminate unnecessary confounding variability is recognized. For MRI, the multi-sequence imaging protocol was very difficult to standardize. At the same time, we thought these results were unusual and that the radiomics analysis could be based on the types of MRI sequences. However, our results showed that the diagnostic performance had no difference among the different sequences, similar to those presented by Hu et al. (41). It is easier to standardize a single MRI sequence than multiple sequences. Hence, we selected the higher value of the AUC for the built Radscore so that the radiomics-based single sequence could optimize the process.

Zhang et al. studied a larger sample size (40). At the same time, we found no significant difference between the single Radscore-based single sequence. The most important characteristic was that we used the images of patients with ASPs before the patient experienced a TIA of stroke. We tried to establish a non-invasive method to predict ASP. In our study, the combined model showed significant differences compared to the clinical model in terms of the Radscore; however, this was not seen in the test group. Zhang et al. showed similar results in the test cohort but not in the training cohort (40). Huang et al. used radiomics-based ultrasonography to non-invasively predict the SPs and ASPs with 0.930 in the training group and 0.922 in the test group, which was higher than that achieved in our study. Meanwhile, they also tried to extract radiomics data from grayscale and strain elasticity images (42). At the same time, the clinical parameters finally included in the combined model were IPH, LRNC, and LHR. Larger studies have shown that IPH and LRNC are high-risk factors for unstable plaques, especially in patients with secondary stroke. The results of the included clinical factors were similar to the results of Zhang's study (40). The difference is that this group of studies included LHR factors as a combined model. The LHR is equal to the ratio of LDL to HDL (43). From the clinical information, it can be seen that the LHR of patients with SP was significantly higher than that of patients with ASP (*p*=0.000). Previous studies have shown that LDL and HDL are risk factors for patients with carotid plaques. Unlike the results of most radiomics studies (44), there is no significant difference between our Radscore and clinical models in terms of diagnostic performance. We believe that radiomics is not suitable for the individual assessment of diseases under the current development, and the combination of clinical parameters can improve the specificity of the diagnostic model. Our final results also show that the combination of Radscore and clinical features can better and effectively improve the diagnostic performance.

In our research, we focused on patients with 30–70% carotid artery stenosis. First, according to the Chinese Guidelines for Carotid Endarterectomy, patients with carotid stenosis ≥70% of ASP and SP should prioritize carotid endarterectomy. At the same time, patients with carotid stenosis <70% of ASP who underwent CEA showed no significant difference compared to the patients with carotid stenosis <70% of ASP who did not undergo CEA (45, 46). Third, research increasingly shows that patients with 70–99% stenosis had a significantly greater 5-year ipsilateral stroke risk than those under <70% stenosis. Research showed that the risk of stroke was consistently 2–3 times higher distal to 70–99% stenosis than to 50–69% stenosis. Compared with 70–99% stenosis, 50–69% stenosis is not a high risk and major factor for SP (10). Lastly, our research focused on 30–70% stenosis in patients of ASP and SP who may consider CEA or carotid artery stenting (CAS). For ≥70% carotid stenosis, stenosis is the major factor for SP but not <70%. Hence, we attempted to build a quantitative imaging biomarker to identify ASP and SP under 70% carotid stenosis.

We built a radiomics signature based on three signal sequence (T1WI, T2WI, PDWI); our results showed that the diagnostic performance showed no difference among the different sequences (Supplementary Material 1). We have used a single T2WI_radscore to combine the clinical variable construct model. Lambin reported the importance of using standardized imaging protocols to eliminate unnecessary confounding variability (15). Multiple sequences of MRI would improve the difficulty of normalizing imaging protocols; thus, we tried to build a Radscore-based single sequence.

Artificial intelligence (AI) and machine learning (ML)-based radiomic analysis is the current trend for precision medicine. Deep learning (DL) and ML-based medical images could be used to build a risk model, diagnose molecular disease subtypes, and predict the response to treatment and the survival year. DL included three steps for prediction: imaging, deep feature extraction, and building model. ML included seven steps for prediction: imaging, image preprocessing, ROI segmentation, hand-crafted feature extraction, feature normalization, feature selection, and building model. DL and ML both have advantages and disadvantages in a clinical decision. At first, ML would be considered as follows: (1) small sample size without hundreds or even thousands, (2) multiple clinical data, and (3) the purpose of prediction is a continuous variable. During the study of ML, image preprocessing, feature robustness, and external validation schemes should be provided to avoid overfitting. In our study, the sample size was small; thus, we chose ML to differentiate the high SPs. However, compared with ML, DL without hand-crafted ROI segementation could improve the robustness of the model. DL has a higher generalizability than ML. The model architecture of DL could be modified such that ML must maintain the same method to provide the feature robustness even under unsupervised training (47). In our study, we chose a single sequence for ML. However, multimodality-based images showed more medical information for clinical decision-making. An example is the difference in diagnostic performance among CT, US, and MRI or the method of optimally combining multiple images to build the best prediction model. DL would be the method considered without pre-processing. Until now, DL architecture exists to aid in clinical decision.

Insufficient data limit the development of DL (48). LASSO can be used because of overfitting and bias. LASSO is suitable for regression of high-dimensional data. The characteristics of LASSO regression are variable selection and regularization, while fitting a generalized linear model. Therefore, irrespective of whether the target-dependent variable (dependent/response variable) is continuous, binary, or multiple discrete, LASSO regression modeling can be utilized for identification. In this study, stepwise variable selection was used to identify an optimal matrix. Complexity adjustment refers to controlling the complexity of the model through a series of parameters to avoid overfitting. For linear models, complexity is directly related to the number of variables in the model. The more variables, the higher is the model complexity. A large number of variables could provide an improved model when fitting, but also poses the risk of overfitting. Typically, when the number of variables is greater than the number of data points or a discrete variable has too many unique values, it is possible to overfit. The degree of LASSO regression complexity adjustment was controlled by parameter λ. The larger the λ, the greater the penalty for the linear model with more variables. Finally, a model with fewer variables is obtained.

This study had several limitations. First, the sample size was small, especially without an external validation group because of the complexity of the parameters in MR imaging that the images improve the difficulty. Second, an increasing number of studies focus on auto segmentation, while we used manual segmentation for extraction of features. Third, there were no external validation groups to provide the generalizability for our model; hence, a large number of sample and multi-center data should be incorporated in future studies. We did not conduct the multiple sequence radiomics analysis to avoid waste of medical resources.

In conclusion, we built a novel model combining radiomics features and clinical risk factors for the identification of symptomatic carotid atherosclerotic plaques. The diagnostic performance of the radiomics model was not significantly different from that of the clinical model. Interestingly, we found that the combined model (Radscore and clinical risk factor) can significantly improve diagnostic performance.

## Data Availability Statement

The raw data supporting the conclusions of this article will be made available by the authors, without undue reservation.

## Ethics Statement

The studies involving human participants were reviewed and approved by the Ethics Committee of Renmin Hospital of Wuhan University. The patients/participants provided their written informed consent to participate in this study.

## Author Contributions

SC and YZ contributed to the conception of the study, performed the data analyses, and wrote the manuscript. SC, CL, and XC performed the experiment. SC, YZ, and WL contributed significantly to analysis and manuscript preparation. LM helped perform the analysis with constructive discussions. All authors contributed to the article and approved the submitted version.

## Funding

This study was financially supported by the National Natural Science Fund Project, China (No. 81871332) and the National Natural Science Youth Project, China (No. 82171895).

## Conflict of Interest

WL was employed by the company GE healthcare. LM was employed by the He Kang corporate Management (SH) Co. Ltd. The remaining authors declare that the research was conducted in the absence of any commercial of financial relationships that could be construed as a potential conflict of interest.

## Publisher's Note

All claims expressed in this article are solely those of the authors and do not necessarily represent those of their affiliated organizations, or those of the publisher, the editors and the reviewers. Any product that may be evaluated in this article, or claim that may be made by its manufacturer, is not guaranteed or endorsed by the publisher.
