# Supplementary material for: A Radiomics Approach to Assess High Risk Carotid Plaques: A Non-invasive Imaging Biomarker, Retrospective Study
Source: Front Neurol. 2022 Mar 8;13:788652. doi: 10.3389/fneur.2022.788652 (PMC8957977; doi:10.3389/fneur.2022.788652)
Supplement: Supplementary file 1 [file Data_Sheet_1.zip › supplement material 3.docx]

Supplementary Material 3

# 1. Radscore based on T1WI and PDWI

# Radscore_T1WI has been built based on T1WI (logλ=) and Radscore_PDWI based on PDWI (logλ=0.043) (Figure #1). The equation of the radiomics signature based on T1WI and PDWI is as follows:


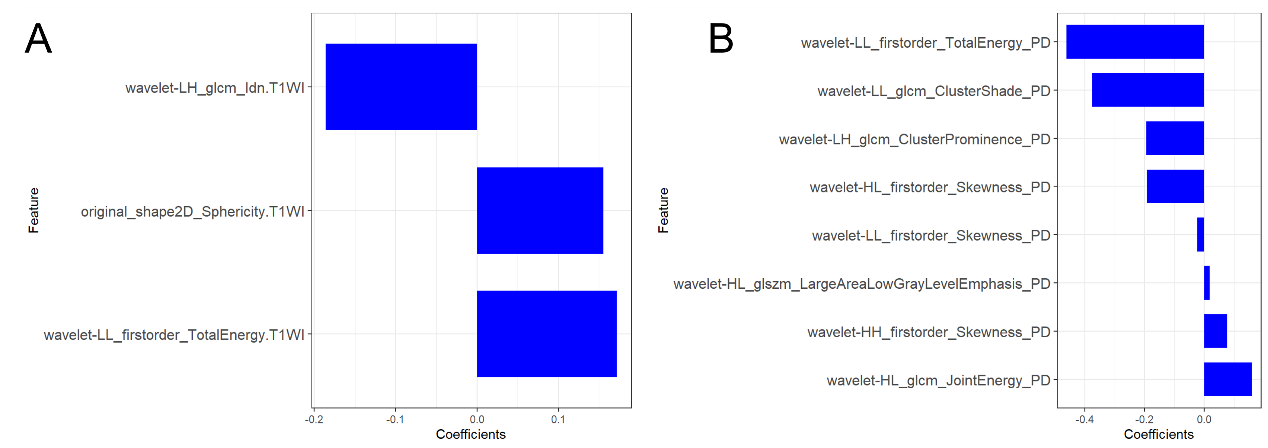


**Supplementary Figure 1.** The coefficient of the features to build the radiomics signature based T1WI (A) and PDWI (B).

$$Radscore\_T1WI=-0.186\times wavelet\_LH\_glcm\_Idn.T1WI+0.172\times wavelet\_LL\_firstorder\_TotalEnergy.T1WI+0.156\times original\_shape2D\_Sphericity.T1WI + 0.65$$

$$Radscore\_PDWI=-0.462\times wavelet\_LL\_firstorder\_TotalEnergy.\_PD-0.376\times wavelet\_LL\_glcm\_ClusterShade.PD-0.193\times wavelet\_LH\_glcm\_ClusterProminence.PD+0.159\times wavelet\_HL\_glcm\_JointEnergy.PD+0.076\times wavelet\_HH\_firstorder\_Skewness.PD-0.192\times wavelet\_HL\_firstorder\_Skewness.PD-0.023\times wavelet\_LL\_firstorder\_Skewness.PD+0.018\times wavelet\_HL\_glszm\_LargeAreaLowGrayLevelEmphasis.PD + 0.658$$

The diagnostic performance of the radscore-based T1WI and PDWI by ROC analysis. The AUC of the radscore based T1WI and PDWI were 0.766 (Supplementary Figure 2A) and 0.790 (Supplementary Figure 2C), respectively, in the training group. The AUC of the radscore-based T1WI and PDWI were 0.750 (Supplementary Figure 2B) and 0.776 (Supplementary Figure 2D), respectively, in the test group, and were lower than the radscore-based T2WI in the training group (AUC=0.834) and test group (AUC=0.818).


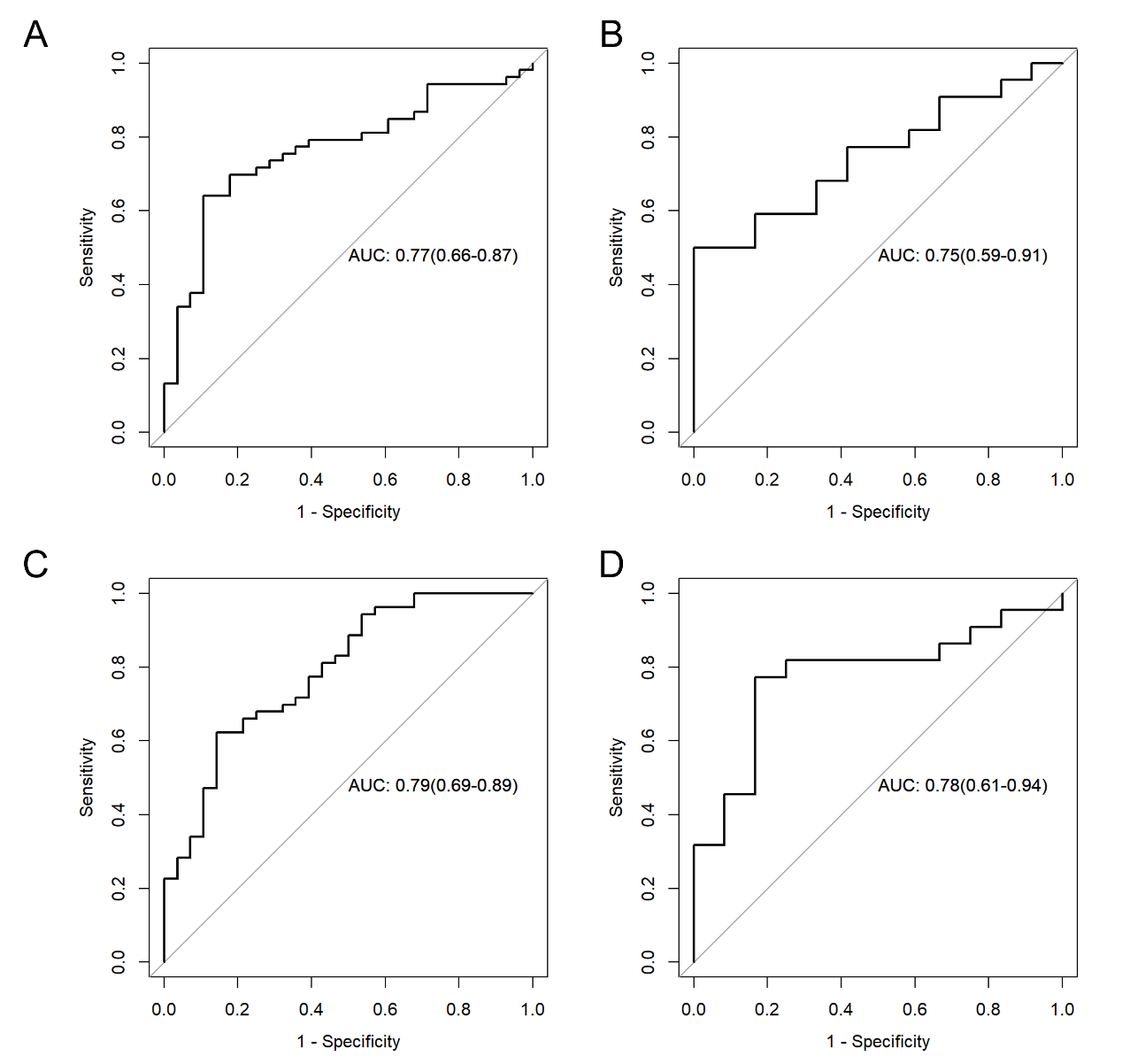


**Supplementary Figure 2.** The ROC analysis of the radscore based T1WI and PDWI in the training group and test group. A&B, Radscore_T1WI in training group and test group. C&D, Radscore_PDWI in training group and test group.

The Delong test was employed to analyze the difference in the diagnostic performance for the radscore in the training group and test group. There were no significant differences between Radscore_T2WI and Radscore_T1WI in the training group (P=0.349) and test group (P=0.502). At the same time, there were no significant differences between Radscore_T2WI and Radscore_PDWI in the training (P=0.492) and test groups (P=0.731). The importance of using standardized imaging protocols to eliminate unnecessary confounding variability is recognized. For MRI, the multi-sequence imaging protocol was very difficult to standardize. At same time, we thought these results were unusual, that the radiomics analysis could be based on the types of MRI sequences. However, our results showed that the diagnostic performance was not different among the different sequences, similar to the study by Hu et al.^1^. It is easier to standardize a single MRI sequence than multiple sequences. Hence, we selected the higher value of the AUC for building the radscore so that the radiomics based single sequence could optimize the process.
